# Hypertension in children in emergency department

**DOI:** 10.15171/jrip.2016.36

**Published:** 2016-08-13

**Authors:** Abdolghader Pakniyat, Parsa Yousefichaijan, Ramin Parvizrad, Morteza Qaribi

**Affiliations:** ^1^Student Research Committee, Emergency Medicine Department, Arak University of Medical Sciences, Arak, Iran; ^2^Associated Professor, Department of Pediatric Nephrology, Arak University of Medical Sciences, Arak, Iran; ^3^Assistant Professor, Department of Emergency Medicine, School of Medicine, Arak University of Medical Sciences, Arak, Iran

**Keywords:** Blood pressure, Pediatric, Emergency medicine

**Implication for health policy/practice/research/medical education:**

Pediatric hypertension is increasing and the incidence of hypertension in the pediatric population. Although reading blood pressure for children is difficult, but it is not be missed by emergency physicians. The emergency physician must certify adequate follow-up for asymptomatic mild hypertension without end organ damage. In contrast hypertension crisis needs evaluation and initiation of treatment in the emergency departments and blood pressure reduction should be performed before the cause of the hypertension is known.

## Introduction

Pediatric hypertension is increasing due to high salt intake, childhood obesity, decrease physical activity, and hyperlipidemia. The incidence of hypertension in the pediatric population, assumed to be 2.5% to 5% (1). According to national consensus statement guideline (2004) a child with three or more blood pressure measurements above the 95th percentile for age, gender, and height should be considered hypertensive (2). Appropriate blood pressure cuff size and standard blood pressure nomograms is essential for accurate measurement of blood pressure levels. Oscillometric device may be used for blood pressure measurement, hence sometimes sphygmomanometer readings is difficult in emergency department (ED), particularly in small child but any abnormal reading should be repeated by auscultation. Although abnormal blood pressure reading is not uncommon due to children are stressed and agitated because of underlying illness or unfamiliar environment, but if repeated blood pressure level remain elevated, careful assessment and treatment should be performed (3,4).

Blood pressure is determined by both peripheral vascular resistance and cardiac output. Factors such as volume overload or sympathetic stimulation by tumors, renin-angiotensin system, drugs, or other processes, may have a main role in the development of hypertension (2,5). Essential hypertension presented with unspecific sign and symptom consist of headache, chest pain, falling asleep, daytime tiredness, and abdominal pain, also oral contraceptives, steroids, and illicit drugs (e.g., cocaine, amphetamines) should be asked in >10 years old children (6,7). The physician must perform a brief but through history and physical examination for the patients with persistently high blood pressure at ED. In the cases with mild to moderate hypertension without any concerning finding, it is recommended to giving educational guides about lifestyle modification and then refer the patients for fallow up with their family physician (1,8).

**Table 1:** Antihypertensive drug agents used in treatment of hypertensive crisis in children 1 to 17 years old (1,8)

**Drug**	**Dosage**	**Route**	**Onset of action**	**Duration**	**Comment**
Labetalol	Bolus: 0.2–1.0 mg/kg/dose, maximum : 40 mg/dose, infusion: 0.25–3.0 mg/kg/h	IV bolus or infusion	5-10 min	2-4 h	Contraindications: asthma, chronic lung disease, heart failure. May mask hypoglycemic symptoms.
Nicardipine	0.5–3.0 μg/kg/min	IV infusion	2-5 min	30-60 min	May cause increased intracranial pressure, headache, nausea, and hypotension.
Hydralazine	0.1–0.5 mg/kg/dose; maximum: 20 mg/dose	IV, IM	10-30 min	4-12 h	Administer every 4 h when given as IV bolus. Not as strong as other agents. Recommended dose is less than U.S. Food and Drug Administration–approved label.
Sodium nitroprusside	0.3–8.0 μg/kg/min	IV infusion	Second	During infusion only	Increase intracranial pressure. Monitor cyanide and thiocyanate levels for patients with renal and liver disease when administering for >24–48 h.
Esmolol	100–500 μg/kg/min (initial dose), then 50–300 μg/kg/min	IV	Seconds	10-20 min	May cause bronchospasm, congestive heart failure, and profound bradycardia.

**Table 2:** Antihypertensive drug agents used in treatment of Hypertensive urgency in Children 1 to 17 Years old (1,8)

**Drug**	**Dosage**	**Route**	**Comments**
Nifedipine	0.1–0.25 mg/kg/dose	PO, sublingual	Precipitous drop in blood pressure, tachycardia, headache.
Minoxidil	0.1–2 mg/kg/dose	PO	Pericardial effusion
Isradipine	0.05–0.1 mg/kg/dose up to 5 mg/dose	PO	Tachycardia, headache
Clonidine	0.05–0.3 mg	PO	Rebound hypertension, sedation

In cases with sever hypertension a careful history and physical examination must be performed. an appropriate history include frequency of urinary tract infections, dysuria, hematuria, frequency, unexplained fevers, edema, history of umbilical artery catheterization as neonate, history of head trauma, ingestion of illicit drugs, oral contraceptives, rapid withdrawal of hypertension drug agents, and history of flushing, sweating, fever, weight loss. In the physical examination should paying a particular attention to the cardiovascular system, neurological and renal system such as four-limb blood pressures, Heart rate, respiratory rate, heart sound, lung sound, oxygen saturation, funduscopic examination, neurologic examination, auscultation abdomen (4,5,7).

Evaluation of renal function, bone marrow response, pregnancy in pubertal girl, and urine drug screening is recommended in ED. The necessity imaging in patient with hypertension in ED are; (*a*) chest x-ray, (*b*) ECG, (*c*) renal ultrasonography, and (*d*) echocardiography. Other imagings including renal arteriography, and brain CT-scan are dictated by the clinical features (1,8).

Primary survey consists of evaluation and stabilization of the air way, breathing, and circulation must be performed for all children with severe hypertension (1). In cases with hypertension crisis cardiac monitoring, placement of Foley catheter is necessary and an arterial catheter is preferable for continuous blood pressure readings. The blood pressure must not reduce more than 25% over 8 hours and it is recommended to gradual reduction of blood pressure to normal valve over the next 26 to 48 hours. Ischemic complications such as renal injury, acute neurologic issues, and blindness are result of too aggressively reducing blood pressure. Hypertension crisis must be treated with intravenous antihypertensive drug agents which have rapid onset of action and short half-life, thus oral medication should be avoided. Medications should be chosen according to their side effect profile, availability, and physician familiarity, hence it is difficult to recommend one particular type of drug over another. Although nephrologists prescribe short-acting nifedipine to treat moderate to severe hypertension in setting of primary renal disorder but there are reports of adverse neurologic events due to rebound hypertension. Oral nifedipine is contraindicated in patients with hypertension crisis, such as intracerebral bleeding, because of the inability to control the amount of blood pressure reduction (Tables 1 and 2) (1,9-12).

Hypertensive urgency may be treated with oral antihypertensive agents. Patients with mild to moderate hypertension in the ED are discharged with instructions for follow-up for outpatient evaluation and treatment (1,8).

It is not unusual that a child presenting to the ED with elevation in blood pressure levels. Although reading blood pressure for children is difficult, but it is not be missed by emergency physicians. Children presenting to the ED maybe have hypertension, it is reasonable to repeat blood pressure level reading after patients rest or acclimation to the environment. Occasionally, however, the elevation in blood pressure levels will be sustained.

## Conclusion

The emergency physician must certify adequate follow-up for asymptomatic mild hypertension without end organ damage. In contrast, hypertension crisis needs evaluation and initiation of treatment in the ED and blood pressure reduction must be performed before the cause of the hypertension is known. Lifestyle modification for obesity, low-salt diet, exercise and avoiding stress is effective for treatment of hypertension, particularly if it is begun from childhood.

## Conflicts of interest

The authors declared no competing interests.

## Authors’ contribution

All authors contributed equally and signed the manuscript.

## Ethical considerations

Ethical issues (including plagiarism, data fabrication, double publication) have been completely observed by the authors.

## Funding/Support

None.
